# Artificial Intelligence Techniques to Optimize the EDC/NHS-Mediated Immobilization of Cellulase on Eudragit L-100

**DOI:** 10.3390/ijms13077952

**Published:** 2012-06-26

**Authors:** Yu Zhang, Jing-Liang Xu, Zhen-Hong Yuan, Wei Qi, Yun-Yun Liu, Min-Chao He

**Affiliations:** Key Laboratory of Renewable Energy and Gas Hydrate & Guangzhou Institute of Energy Conversion, Chinese Academy of Sciences, Guangzhou 510640, China; E-Mails: zhangyu@ms.giec.ac.cn (Y.Z.); yuanzh@ms.giec.ac.cn (Z.-H.Y.); qiwei@ms.giec.ac.cn (W.Q.); liuyun@ms.giec.ac.cn (Y.-Y.L.); hemc@ms.giec.ac.cn (M.-C.H.)

**Keywords:** immobilized enzyme, cellulase, artificial intelligence based optimization, smart biocatalyst, carbodiimide coupling

## Abstract

Two artificial intelligence techniques, namely artificial neural network (ANN) and genetic algorithm (GA) were combined to be used as a tool for optimizing the covalent immobilization of cellulase on a smart polymer, Eudragit L-100. 1-Ethyl-3-(3-dimethyllaminopropyl) carbodiimide (EDC) concentration, *N*-hydroxysuccinimide (NHS) concentration and coupling time were taken as independent variables, and immobilization efficiency was taken as the response. The data of the central composite design were used to train ANN by back-propagation algorithm, and the result showed that the trained ANN fitted the data accurately (correlation coefficient *R^2^* = 0.99). Then a maximum immobilization efficiency of 88.76% was searched by genetic algorithm at a EDC concentration of 0.44%, NHS concentration of 0.37% and a coupling time of 2.22 h, where the experimental value was 87.97 ± 6.45%. The application of ANN based optimization by GA is quite successful.

## 1. Introduction

Cellulase plays an important role in the conversion of lignocellulosic biomass to biochemicals, biomaterials and bioenergy. In order to recover the enzyme after reaction for possible re-use, cellulase has always been immobilized on insoluble matrices as opposed to soluble matrices in the past. However, poor contact during reaction is a consequence of such an immobilization [1–4], especially when an insoluble cellulosic biomass was used as the enzymatic substrate [5]. Using S-IS (soluble-insoluble) matrices to immobilize cellulase could help in this respect by providing a smart biocatalyst [6,7]. The smart biocatalyst is not only a homogeneous catalyst for reducing mass transfer resistance during the reaction, but also a heterogeneous catalyst for easy recovery after the reaction.

**Figure 1:**
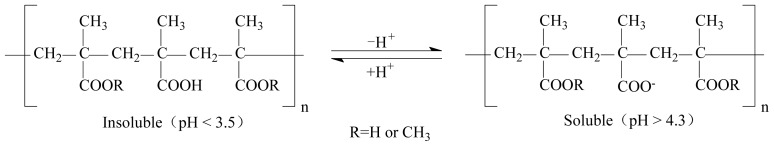
The chemical structure and reversible soluble mechanism of Eudragit L-100.

As a common smart polymer, Eudragit L-100 has been used to immobilize cellulase by carbodiimide coupling. Eudragit L-100 is a copolymer of methacrylic acid and methyl methacrylate, and contains many carboxyl groups (Figure 1). Carbodiimide was able to activate the carboxyl groups, and then cellulase was bonded to the activated Eudragit L-100. However, the immobilization is non-covalent due to the existence of acetate (acetate also contains many carboxyl groups), therefore, the activity yield was relatively low and the reusability was unsatisfactory [8]. To address this the problem, cellulase was immobilized on Eudragit L-100 in the absence of acetate, and *N*-hydroxysuccinimide (NHS) was added to enhance the carbodiimide coupling [9,10]. However, our preliminary experiments showed that immobilized cellulase with a high activity (for filter paper) did not show a correspondingly strong ability to hydrolyze lignocellulosic biomass such as straw, grass and wood. This may be attributed to the structure and composition difference of enzymatic substrates. Lignocellulosic biomass consists of lignin, cellulose and hemicellulose, while filter paper is just like pure cellulose. In a practical application, the enzymatic substrate is a lignocellulosic biomass and not pure cellulose. So in this study, glucose produced from the hydrolysis of a lignocellulosic biomass by immobilized cellulase was used as the response to optimize the immobilization conditions. The more glucose was produced, the larger the hydrolytic ability of immobilized cellulase and the higher the immobilization efficiency.

Model based optimization techniques have been gaining much popularity because they cannot only examine the combined interaction of each factor, but also are labor-saving compared to one-factor-at-a-time approaches. Response surface methodology (RSM) is such a frequently used model and has achieved much progress in optimizing multi-factor process, especially for three-factor processes [11,12]. However, RSM has a limitation in simulating the data of an irregular experimental domain and can only exhibit a low-order non-linear behavior to a regular experimental region. In contrast, another model, namely artificial neural network (ANN), does not suffer from the limitation of the experimental design, and the efficient simulation requires relatively fewer experiments [13]. Recently, ANN showed a significantly higher simulation and prediction accuracy than RSM in simulating and predicting many biochemical reactions [9,14–21]. Moreover, a higher and more accurate optimized value is always obtained from ANN (combined with genetic algorithm (GA)) than RSM [9,15,17–19,21,22]. GA is another artificial intelligence tool that uses evolutionary natural selection processes, where selection results in species that fit the best.

Like our previous report [9], two artificial intelligence techniques (ANN and GA) were used to optimize cellulase immobilization.

## 2. Results and Discussion

### 2.1. ANN based Simulation and Prediction

After limited trials, the training goal was achieved, and the ANN was built successfully. The fitted immobilization efficiency by ANN is listed in Table 1. The table shows that the experimental values were almost identical to the fitted values. The mean absolute/relative error, root-mean-square error and variance that were used to evaluate the ANN based simulation performance were 0.74, 1.18%, 0.99 and 0.98, respectively. The values are very smaller, which also shows that the fit accuracy is very high. Similar results were obtained for other bioprocesses when using ANN based fit [23–25]. The analysis of variance (ANOVA) is given in Table 2. According to the *F*-value and *P*-value, it is outlined that the ANN is a significant model. The correlation coefficient of the two sets of data (experimental and simulated values) is more than 0.99. The value is very close to 1, which further demonstrates that the fit is rather perfect.

In order to validate the trained ANN, three more experiments were carried out (trial 21–23 in Table 1). The result shows that the experimental values are rather close to the ANN based prediction. All the relative errors between experimental and predicted values are within 3.0%. Both fit and prediction results show the training of the ANN is quite successful. So, the trained ANN could be considered as the desirability function between immobilization efficiency and the three factors.

### 2.2. ANN based Optimization by GA

**Figure 2:**
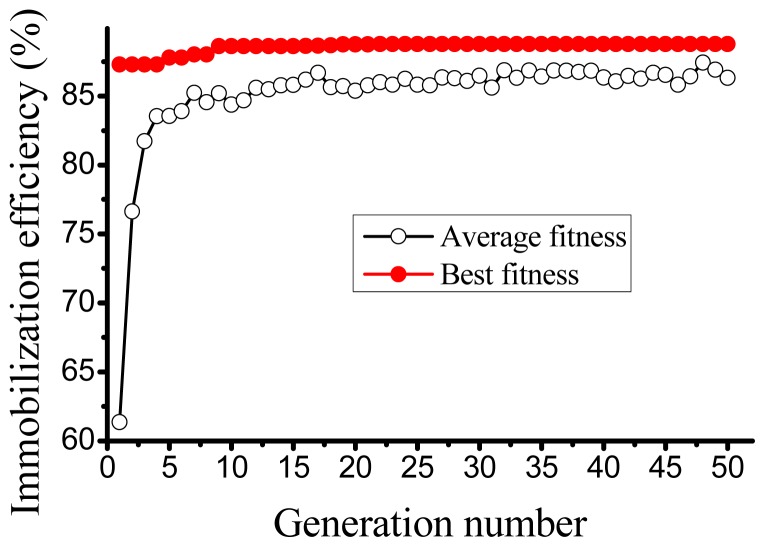
Evolution of the best and average fitness (immobilization efficiency) over the 50 generations in the genetic algorithm.

Once the ANN was built successfully, GA was used to search the maximum output. Results of 50 stochastic runs show that the range of maximum, minimum and average objective function is from 84.23% to 89.76%, from 40.28% to 48.96% and from 78.23% to 80.99%, respectively. The average value of maximum objective function is calculated as 88.76%, and the value can be considered as the optimized value by artificial intelligence techniques (ANN-GA). Correspondingly, the optimized condition is a EDC concentration of 0.44%, a NHS concentration of 0.37% and a coupling time of 2.22 h, where the experimentally determined immobilization efficiency was 87.97 ± 6.45%. This shows a perfect agreement with the ANN based optimization (less than 1% derivation). Similar results were obtained for other bioprocesses [24,26,27]. Figure 2 shows the evolution of the algorithm with successive generations. Starting from 61.36%, the average immobilization efficiency apparently increases until the 7th generation and is 86.33% at the end of 50 generations. The maximum immobilization efficiency also apparently increases for the first few generations and reached 88.76% at the 22th generation, then remains unchanged.

Compared to frequently used RSM, artificial intelligence represents superior non-linearity, more accurate simulation and prediction, so a better optimization could always be obtained [28]. Besides, artificial intelligence does not suffer from the limitation of experimental design, and the efficient simulation requires relative fewer experiments. Of course, the accuracy would be higher when a large number of experiments are used to create the non-linear behavior [29]. Thus, in case of artificial intelligence, a more liberal search space can be chosen, although the correlation in that search space is more complex than the equation of higher degree [30].

### 2.3. Reusability

Immobilized cellulase was mixed with insoluble substrate at stirring. After the reaction, the undegraded substrate was filtered or precipitated by centrifugation. Then, the pH of the obtained supernatant was lowered and centrifuged. The obtained precipitation was the recycled immobilized cellulase, which could be used for the next hydrolysis. As our previous reports [9,10] state, there is more than 50% productivity after five re-uses.

## 3. Experimental Section

### 3.1. Materials

Eudragit L-100 was obtained from Degussa Ltd. (Shenzhen, China). The polymer is completely soluble at pH > 4.3 in aqueous solution, and the critical soluble pH changes to 5.0 via coupling with cellulase (Figure 1) [8]. EDC and NHS were purchased from Sigma-Aldrich Co., Ltd. (Shanghai, China). Crude cellulase powder from *Trichoderma viride* was provided by Shanghai Bio Life Science & Technology Co., Ltd. (Shanghai, China). The activity is 74.07 FPU/g (FPU is the activity unit of cellulase when filter paper is used as the enzymatic substrate), assayed by the description of IUPAC [31]. Wheat straw was obtained from a local farm and pretreated by alkali as Carrillo *et al.* described [32].

### 3.2. Immobilization of Cellulase on Eudragit L-100

Cellulase was covalently immobilized on Eudragit L-100 by carbodiimide coupling in the presence of NHS following protocol. Fifty milliliters of solution of Eudragit L-100 (2%, w/v) was prepared as Sardar *et al.* described [33]. To activate the polymer, NHS (0.08%–0.48%, w/v) and EDC (0.06%–0.74%, w/v) were added in turn. After mixing for 15 min, some crude cellulase containing 100 mg protein was added and stirred for 0.48 to 5.52 h. The choice of time range was based on our previous study, where the optimum coupling time in the absence of NHS was about 3 h. The pH of the mixture was reduced to 3.6 with glacial acetic acid. Precipitates were separated by centrifugation (6800 × *g*, 10 min) at 4.0 °C and washed three times with 0.02 mol/L acetic acid. At last, the precipitations were re-dissolved in 50 mL acetate buffers (0.2 mol/L, pH 5.0) and used as immobilized cellulase for further hydrolytic experiments.

### 3.3. Central Composite Design

Eudragit L-100 is a copolymer of methacrylic acid and methyl methacrylate, which contains many carboxyl groups. These carboxyl groups are inevitably used as preferred functional groups to couple cellulase. EDC could help with this. EDC is generally utilized as a carboxyl-activating agent for amide bonding with primary amines and NHS could enhance the coupling. Besides EDC (coupling agent) and NHS (enhancer), the coupling time is also an important factor that can affect the coupling between Eudragit L-100 and cellulase. Less time can result in the cellulase not being coupled to Eudragit L-100 in time; more time might bring an excess coupling that negatively affects the active site of cellulase. So in this study, EDC concentration X_1_, NHS concentration X_2_ and coupling time X_3_ were applied as independent variables (inputs of ANN). A central composite design (CCD) for the three factors was applied to train ANN. The range and levels of each factor is shown in Table 3. The CCD with 20 trials (six central points) was a 2^3^ full factorial design at a distance 1.68 from the origin (Trials 1–20 in Table 1).

### 3.4. Artificial Neural Network

**Figure 3:**
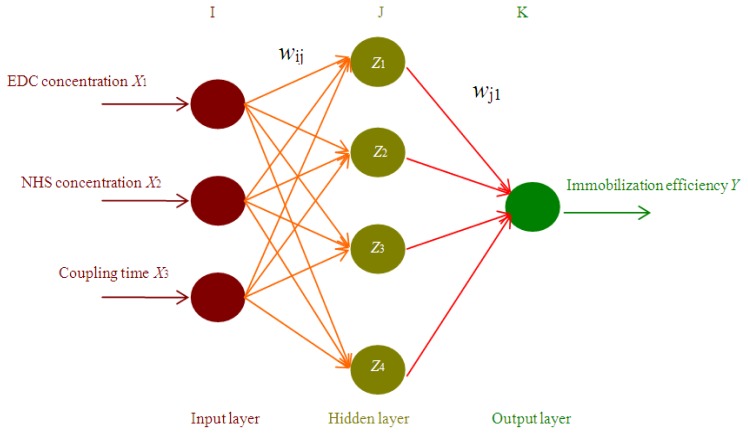
Schematic representation of ANN modeling the relationship between immobilization efficiency and three factors (EDC concentration, NHS concentration and coupling time). The factors *w*_ij_ and *w*_j1_ are the connecting weights from *X*_i_* to *Z*_j_ and *Z*_j_ to Y*, respectively.

ANN is a computer program architecture capable of non-linear computations in certain configurations, such as the multi-layer perceptron (MLP). It can identify arbitrary discriminant functions directly from experimental data [28,34]. In our experiment, the ANN architecture consists of three neurons (EDC concentration X_1_, NHS concentration X_2_ and coupling time X_3_) in the input layer, four neurons in the hidden layer, and one neuron (immobilization efficiency) in the output layer (Figure 3). This is a typical neural network architecture [34]. In order to receive equal attention during the training process [26], all the data (input and output ones) of the CCD (trial 1–20 in Table 2) were scaled as follows (Equation (1)):

(1) $$\begin{array}{l} \text{Input layer}:\;\;\;{X_{\text{i}}}^{*}=2\frac{X_{\text{i}}-X_{\text{i},\text{min}}}{X_{\text{i},\text{max}}-X_{\text{i},\text{min}}}-1\\ \text{Output layer}:\;\;\;Y^{*}=\frac{Y-0}{100-0} \end{array}$$

where, *X*_i_* and Y* are the new scaled data of input and output layers.

As the most frequent algorithm, the back-propagation algorithm was used to train a random ANN model by feeding the newly scaled data. The algorithm includes forward propagation of signal and back propagation of error. Forward propagation of signal was carried out as follows: Hidden layer:

(2) $$\begin{array}{c} \text{Hidden layer}:\;Z_{j}=f\left(\sum_{i=1}^{I}w_{ij}X_{i}^{*}\right)\\ \text{Output layer}:\;Y^{*}=f\left(\sum_{j=1}^{J}w_{j}Z_{j}\right) \end{array}$$

where, *Z*_k_ are the data of hidden layer, respectively; *w*_ij_ and *w*_j_ are connecting weights from *X*_i_* to *Z*_j_ and *Z*_j_ to Y*, respectively, and f() is the transfer function.

The transfer functions in the hidden and output layers of the ANN were tangent sigmoid and pure linear functions, respectively. The mean squared error between the results of the output neurons and the actual outputs is calculated and propagated backward through the network. Then the algorithm adjusts the weight of each. Once the mean square error reached 1e–4, the training was over and the corresponding ANN was built. All the procedures were carried out by Matlab 7.1.

### 3.5. Genetic Algorithm

Using the trained ANN as the fitness function, a genetic algorithm (GA) was coupled to search the maximum immobilization efficiency. The objective function is to find a decision variable, *i.e.*, ANN input neurons (*X*_i_), so that it maximizes the objective function, *i.e*., ANN output. Working parameters namely the total number of generations, population size, number of binary coded variables, cross over probability and mutation probability are 50, 20, 3, 0.4 and 0.005, respectively.

Genetic algorithm uses evolutionary natural selection processes, where selection results in species that fit the best. A population of individuals is maintained at each generation, and each individual in the population represents a possible solution to the problem [27]. The individual chosen in this study was a set of EDC concentration *X*_1_, NHS concentration *X*_2_ and coupling time *X*_3_. The GA-based search for an optimal solution vector, *X*_i_, begins with a randomly initialized population of probable (candidate) solutions. The candidates are referred to as strings or chromosomes. Each chromosome is evaluated to measure its fitness using the ANN-based model. The steps involved in GA-based optimization algorithm are as follows:

Randomly generate a population of individuals and assign a fitness value to each individual to guide the search by specific fitness function. Select individuals with higher fitness values and let them undergo genetic operation, including crossover and mutation. Use the newly generated child population as the parent population for the next generation and treat them with the same evolutional process continuously until a stop criterion has been satisfied [20,27]. The algorithm was run 50 times in this study. All the procedures were carried out by Matlab 7.1.

### 3.6. Determination of Immobilization Efficiency

Before and after immobilization, 100 mg cellulase protein was incubated with 2.5 g pretreated wheat straw at pH 5.0, 50 °C and 120 rpm. The solid loading was 5% (v/w). After 12 h, a sample solution was taken out, and centrifuged at 4000 rpm and 4 °C for 5 min. The obtained supernatant was kept at 80 °C for 10 min and then used for glucose assay. Immobilization efficiency was calculated as follows:

(3) $$\text{Immobilization efficiency }(\%)=\frac{\text{Glucose produced by immobilized cellulase}}{\text{Glucose produced by free cellulase}}\times 100$$

### 3.7. HPLC Method

Glucose was determined by the HPLC Waters 2695 system consisting of Waters 600E system controller, Waters 717 automatic sampler, Waters 2414 differential refractometer, Shodex sugar SP-0810 column. The mobile phase was distilled water at a flow rate of 0.6 mL/min. The column temperature was 80 °C. The injected sample volume was 10 μL. Standard samples and hydrolyzed samples were filtrated by a 0.45 μm filter before analysis.

## 4. Conclusions

Cellulase, via immobilization, was converted to a smart biocatalyst that could be used as a homogeneous catalyst during the reaction and recovered easily after the reaction for possible re-use. It is self-evident that the immobilization could improve the economy of cellulase utilization in its related industries. In this study, the artificial intelligence based optimization is quite successful, and 87.97% of immobilization efficiency is obtained. It is believed that artificial intelligence based optimization technique could be applied in more and more complicated biochemical systems due to its advanced non-linear analysis and mechanistic independence shown in modeling and predicting these systems.

## Figures and Tables

**Table 1:** Experimental design matrix of three factors and the experimental immobilization efficiency versus artificial neural network (ANN) simulated/predicted values. Data are means ± SD of triplicates.

Trials	X_1_	X_2_	X_3_	Immobilization efficiency (%)	

				Experimental	ANN
**Data for ANN simulation (CCD)**					
1	−1.00	−1.00	−1.00	76.29 ± 5.25	77.69
2	+1.00	−1.00	−1.00	46.53 ± 3.14	46.85
3	−1.00	+1.00	−1.00	45.64 ± 3.52	45.53
4	+1.00	+1.00	−1.00	67.12 ± 4.94	68.28
5	−1.00	−1.00	+1.00	59.45 ± 4.15	59.58
6	+1.00	−1.00	+1.00	48.80 ± 3.88	49.40
7	−1.00	+1.00	+1.00	61.89 ± 4.68	64.31
8	+1.00	+1.00	+1.00	73.77 ± 5.01	74.10
9	−1.68	0.00	0.00	54.74 ± 4.21	52.89
10	+1.68	0.00	0.00	64.84 ± 4.52	63.87
11	0.00	−1.68	0.00	45.73 ± 3.57	47.18
12	0.00	+1.68	0.00	63.49 ± 4.36	62.75
13	0.00	0.00	−1.68	55.12 ± 3.89	54.17
14	0.00	0.00	+1.68	48.25 ± 3.31	48.00
15	0.00	0.00	0.00	77.01 ± 5.15	76.86
16	0.00	0.00	0.00	77.33 ± 5.07	76.86
17	0.00	0.00	0.00	77.22 ± 5.20	76.86

**Data for ANN simulation (CCD)**					

18	0.00	0.00	0.00	77.06 ± 5.19	76.86
19	0.00	0.00	0.00	77.30 ± 5.09	76.86
20	0.00	0.00	0.00	77.28 ± 5.08	76.86

**Data for ANN prediction (random)**					

21	−1.68	0.00	+1.00	76.22 ± 4.77	78.17
22	−1.00	+1.00	0.00	71.31 ± 3.98	69.38
23	0.00	+1.68	+1.68	57.59 ± 2.56	56.87

**Table 2:** Analysis of variance (ANOVA) for neural network model ANN.

	DF	SS	MS	*F*-value	*p*-value	*R^2^*
Model	1	2909.454	2909.454	2865.166	2.68E–21	0.9938
Residual	18	18.27823	1.015457			
Total	19	2927.732				

**Table 3:** Experimental levels and range of each factor.

Factors	Symbols	Ranges and levels				

		−1.68	−1.00	0	1.00	1.68
EDC concentration	X_1_	0.06	0.20	0.40	0.60	0.74
NHS concentration	X_2_	0.08	0.16	0.28	0.40	0.48
Coupling time	X_3_	0.48	1.50	3.00	4.50	5.52
